# Automatically Identifying Self-Reports of COVID-19 Diagnosis on Twitter: An Annotated Data Set, Deep Neural Network Classifiers, and a Large-Scale Cohort

**DOI:** 10.2196/46484

**Published:** 2023-07-03

**Authors:** Ari Z Klein, Shriya Kunatharaju, Karen O'Connor, Graciela Gonzalez-Hernandez

**Affiliations:** 1 Department of Biostatistics, Epidemiology, and Informatics Perelman School of Medicine University of Pennsylvania Philadelphia, PA United States; 2 Autism Spectrum Program of Excellence Perelman School of Medicine University of Pennsylvania Philadelphia, PA United States; 3 Department of Computational Biomedicine Cedars-Sinai Medical Center West Hollywood, CA United States

**Keywords:** natural language processing, data mining, social media, COVID-19, Twitter

## Introduction

Studies have shown that Twitter can be a complementary source of data for monitoring personal experiences of COVID-19, such as symptoms [1-8]. Given the lack of manually annotated training data for supervised machine learning, however, these studies relied on other methods to identify English-language tweets that self-report a COVID-19 infection, including keywords [1-3], regular expressions [4,5], transfer learning [6], self-supervised learning [7], and unsupervised learning [8]. As Mackey et al [8] suggest, “supervised models that can leverage validated training sets are likely to have a much higher performance… and could likely achieve classification closer to real time.” The objective of this study was to develop and deploy a manually annotated data set and benchmark classification models for automatically identifying users who have self-reported a COVID-19 diagnosis. To validate self-reports of COVID-19 infection, we included only tweets that provide evidence of a diagnosis, such as a positive test, clinical diagnosis, or hospitalization.

## Methods

### Ethical Considerations

The institutional review boards of the University of Pennsylvania and Cedars-Sinai Medical Center reviewed this study and deemed this human subjects research as exempt.

### Data Collection

Between July 2020 and May 2021, we collected approximately 600,000 English-language tweets, excluding retweets, from the Twitter streaming application programming interface (API) that included keywords related to both COVID-19 and a test, diagnosis, or hospitalization as a tokenized match (Multimedia Appendix 1). For tweets that mentioned a test, we also required them to include the keyword *positive*. We then searched these tweets for personal references to the user and automatically excluded tweets with select references to other people who were assumed not to be members of the user’s household. The full query (Multimedia Appendix 2) returned 70,319 tweets that were posted by 58,847 users.

### Annotation

We randomly sampled 10,000 (14%) of the 70,319 tweets, posted by unique users, and developed annotation guidelines (Multimedia Appendix 3) to help 3 annotators distinguish tweets that self-reported a COVID-19 diagnosis from those that did not. Among the 10,000 tweets, 9000 (90%) were annotated by 2 annotators and 1000 (10%) were annotated by all 3 annotators. Interannotator agreement (Fleiss κ), based on these 1000 tweets, was 0.79. After resolving the disagreements among all 10,000 tweets, 1728 (17%) were annotated as self-reporting a COVID-19 diagnosis and 8272 (83%) as not.

### Automatic Classification

We split the 10,000 tweets into 80% and 20% random sets as training data (Multimedia Appendix 4) and held-out test data, respectively, and performed machine learning experiments using 5 deep neural network classifiers based on bidirectional encoder representations from transformers (BERT) [9]. We preprocessed the tweets by normalizing URLs and usernames and lowercasing the text. For training, we used Adam optimization, a batch size of 8, 5 epochs, and a learning rate of 0.00001, based on evaluating models after each epoch using a 5% split of the training set. We fine-tuned all layers of the transformer models with our annotated tweets.

## Results

Table 1 presents the performance of the classifiers. The COVID-Twitter-BERT classifier, based on a BERT model that was pretrained on tweets related to COVID-19 [10], achieved the highest *F*_1_-score: 0.94 (precision=0.96, recall=0.91). We deployed the classifier on 948,859 unlabeled tweets retrieved by our query (Multimedia Appendix 2) through January 2023, and 222,084 of them were detected as self-reports of a COVID-19 diagnosis, posted by 181,521 users (Multimedia Appendix 5). To validate precision over time, we annotated 1500 automatically classified tweets that were posted up to 15 months after our initial data collection, identifying 1451 true positives (precision=0.97).

Table 2 presents examples of false positives and false negatives of the COVID-Twitter-BERT classifier in the test set. Among the 12 false positives, 4 (33%) were reported speech, such as quotations (tweet 1), and 2 (17%) reported a positive antibody test (tweet 2), which were annotated as “positive” when the tweet did not imply that the test result may have been associated with vaccination. Among the 29 false negatives, 11 (38%) reported being hospitalized (tweet 3), 3 (10%) mentioned a negative COVID-19 test (tweet 4), and another 3 (10%) reported receiving treatment for COVID-19 (tweet 5).

**Table 1:** Precision, recall, and *F*_1_-scores of deep neural network classifiers for the class of tweets that self-report a COVID-19 diagnosis, evaluated on a held-out test set of 2000 manually annotated tweets.

Classifier	Precision	Recall	*F*_1_-score
BERT-Base-Uncased	0.82	0.85	0.84
DistilBERT-Base-Uncased	0.83	0.77	0.80
RoBERTa-Large	0.87	0.92	0.90
BERTweet-Large	0.90	0.91	0.91
COVID-Twitter-BERT	0.96	0.91	0.94

**Table 2:** Sample false-positive and false-negative tweets of the COVID-Twitter-BERT classifier (with the keywords that matched the data collection query in italics).

Number	Tweet	Actual	Predicted
1	“*I* am always advocating for people to get the vaccine,“ says @QCC_CUNY Public Safety Specialist Doodnauth Singh. ”It is safe and has been *tested* a lot. *I* am in excellent health, but *tested positive* for *COVID* in December. Stay safe, not sorry.“	–	+
2	*I* just received the results of *my COVID* Antibody *test*. After 6 months from *my* 2nd shot, *I* am happy to report that *I tested POSITIVE*!!!!	–	+
3	After another night *in the hospital I*’ve decided *I* won’t let *Covid* take *me* out! *I*’m Hanging on!	+	–
4	*Me* and *my* bf literally sleep in the same bed everyday his *covid test* was negative mines was *positive* this is crazy 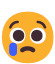	+	–
5	*I*'ve had and recovered from *covid* getting monoclonal antibodies. *I* got the J & J vaccine. *I* read that *I* have a 90% chance of not contracting *covid* again and a 100% chance of not being *hospitalized*. Are these numbers true?	+	–

## Discussion

The benchmark performance of supervised classification demonstrates the utility of our annotated training data (Multimedia Appendix 4) for automatically identifying Twitter users who have self-reported a COVID-19 infection, facilitating the use of Twitter data for monitoring personal experiences of COVID-19 in real time. Although our approach is limited to users who report evidence of a diagnosis, our deployment demonstrates that users can be identified on a large scale (Multimedia Appendix 5).

## Appendix

Multimedia Appendix 1 Twitter API keywords for tokenized tweet matching.

Multimedia Appendix 2 Data collection query.

Multimedia Appendix 3 Annotation guidelines.

Multimedia Appendix 4 Training data.

Multimedia Appendix 5 Large-scale cohort.

## Abbreviations

API: application programming interface
BERT: bidirectional encoder representations from transformers

## Data Availability

The manually annotated training data and unlabeled data resulting from the automatic classification are included with this published article in its supplementary information files, as Multimedia Appendices 4 and 5, respectively. In accordance with the Twitter Terms of Service, these tweets are made available as tweet IDs, which can be rehydrated as tweet objects if they remain public at the time they are requested through the Twitter API.
