# Supplementary material for: Automatically Identifying Self-Reports of COVID-19 Diagnosis on Twitter: An Annotated Data Set, Deep Neural Network Classifiers, and a Large-Scale Cohort
Source: J Med Internet Res. 2023 Jul 3;25:e46484. doi: 10.2196/46484 (PMC10365612; doi:10.2196/46484)
Supplement: Multimedia Appendix 3 [file jmir_v25i1e46484_app3.docx]

COVID-19 Diagnosis Tweet Classification Annotation Guidelines

Table of Contents

[Project Summary 1](#_Toc126934874)

[The Role of Annotation 2](#_Toc126934875)

[Annotation Guidelines 2](#_Toc126934876)

[Annotation Tool 3](#_Toc126934877)

[Annotation 4](#_Toc126934878)

[General Annotation Guidelines 4](#_Toc126934879)

[Positive Cases 5](#_Toc126934880)

[User Self-Report 5](#_Toc126934881)

[Clinical Diagnosis 6](#_Toc126934882)

[Negative Cases 8](#_Toc126934883)

[Negative or Unreported Testing Results 8](#_Toc126934884)

[Report **Not** of User’s Diagnosis 8](#_Toc126934885)

[Clinical Diagnosis not Reported 9](#_Toc126934886)

[Special Cases 9](#_Toc126934887)

[Special Cases – Positive 10](#_Toc126934888)

[Conflicting Test Results Reported 10](#_Toc126934889)

[Special Cases – Negative 10](#_Toc126934890)

[Quoted or Reported Speech 10](#_Toc126934891)

[Positive Antibody Test Due to Vaccine 11](#_Toc126934892)

# Project Summary

For this project, we want to identify Twitter users that report having a clinical diagnosis of COVID-19, which may be expressed in the tweet as being diagnosed or hospitalized with, or testing positive for, COVID-19. Users identified as having COVID-19 will then be part of larger studies to identify their symptoms and the duration of these symptoms, their vaccination status, and their demographic information (age, gender, place of residence)**.**

The annotation needed for this part of the project is a binary classification of whether the tweet from the user contains a mention, or statement, they have been clinically diagnosed with COVID-19. We will use the annotated data to train machine learning (ML) classifiers to automate the identification of users who have stated in Twitter that they were diagnosed with COVID-19.

# The Role of Annotation

Natural language processing (and machine learning in general) requires labeled (annotated) data for use in developing and testing systems. For example, if your system is intended to find tweets that mention that a user has been diagnosed with COVID-19, then to train and test it, you will need some tweets that mention a self-report of a COVID-19 diagnosis —and some tweets that don’t. We will create such data by reading tweets and labelling them with information regarding their contents.

While it is quite easy for us to find tweets that might be relevant using the computer through keyword searches, it is difficult to have the computer find exactly what we are looking for without providing some examples for it to learn from. Part of the reason that this task is much easier for you than it is for a computer is that humans are quite good at something that computers are terrible at: inference. That means that humans can easily draw logical conclusions that are quite difficult for computers in most situations. For example, to find our positive cases we could easily program the computer to find all the tweets that state “I was diagnosed with COVID-19”. However, a system like this would not find the people who do not state their diagnosis in such a direct manner such as, “*Caught Covid, spent two months in the hospital, got trached and now spending my days looking for affordable home care so my kids can go to school and work*.” In this example, the user never states explicitly that they were the one who had COVID by using the first-person pronoun “I”, however, we can infer from the rest of the tweet that they are speaking about their personal experience and are providing a self-report of COVID.

By providing the computer model with human annotated data, the system can “learn” from the examples provided and the model will then be able to classify a much larger set of unlabeled data. In order to create a robust and accurate system, the training data must be consistently annotated in accordance with the annotation guidelines for the project.

# Annotation Guidelines

The guidelines have been developed based on what we envision we will be able to develop and train a ML classifier to detect automatically, namely a person who reports in a tweet that they have been clinically diagnosed with COVID-19. While there may be many ways to define what would constitute a person declaring they had COVID-19 in a tweet, due to our plans to include the users in future studies, we want to ensure, with as much certainty as we can, that the user did have COVID-19. Therefore, we have established certain criteria that must be mentioned in the tweet in order to classify it as a positive case. These guidelines define the constraints we have identified that meet **our** definition of a COVID-19 positive case for the purpose of this study, and those that will follow.

To ensure that the annotation decisions are consistent with our definition. the guidelines define our criteria to be met for each class so the annotators can use that information for their decision-making during annotation. While some examples are given to demonstrate how a particular criterion might be expressed in a tweet, it would be impossible to provide an example for every possible way a user may state their information. In these situations, the annotator will have to use their best judgement using the guidelines and make inferences given the information in the tweet.

The purpose of the guideline is not to tell the annotator how each tweet should be annotated but rather to give them the background knowledge needed to decide if a tweet meets the criteria.

As the annotation project progresses, it may be necessary to make updates or changes to the guideline.

# Annotation Tool

The annotation will be performed using an Excel spreadsheet (Figure 1). The documents for the annotation tasks will be prefilled with the following information: tweet_id, user_id, tweet text and date created.

The annotators must complete the “Class” column, coding each tweet with either a “0” or a “1”.

In addition, there is a ‘Notes’ column. This is where the annotator should place any questions, comments or difficulty encountered with the annotation of the tweet. The “Notes” field is not required to be completed for each tweet, but annotators are encouraged to use it whenever they encounter a tweet that was challenging to annotate. These Notes are important not only for resolving disagreements, but also may provide indications that the guidelines may not be clear, or may not have covered all situations, and need to be updated.

The Annotation section below will define and provide instruction and examples for the annotator for this annotation project.


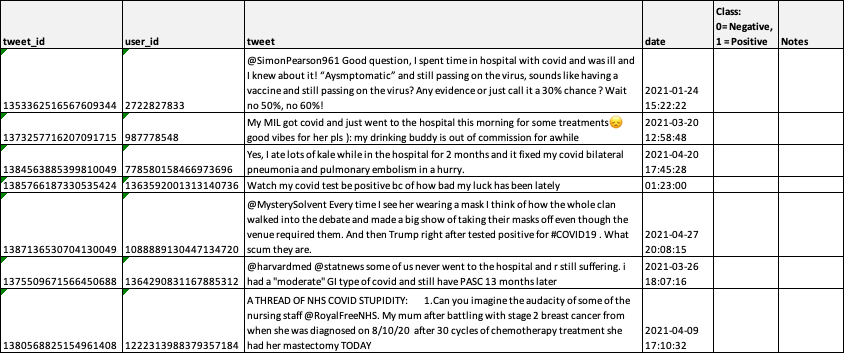


**Figure 1**: Sample of Annotation File

# Annotation

## General Annotation Guidelines

1. For the purpose of this study, **clinically diagnosed** means that the user states in the tweet that they have been “diagnosed with”, “hospitalized for” or “tested positive” for COVID-19 or COVID-19 antibodies. For the sake of brevity, we will use the term “**diagnosed**” throughout the guideline but this term encompasses all three possible conditions.
2. All variants used to describe COVID-19, such as Covid, Corona, Coronavirus etc, are considered equal as mentions of COVID-19. As is the use of COVID as an adjective to describe a disease, such as “COVID pneumonia”. Annotators should consider all as discussions of COVID-19.
3. The diagnosis must be for the user, that is, a self-reported COVID diagnosis
4. Annotators will classify each tweet in the data set as either containing a mention by the user that they have, or have been at some time in the past, diagnosed with COVID-19 (code = “1”), or no indication in the tweet that user was diagnosed with COVID-19 (code = “0”).
5. Each tweet can only be designated with one code (“0” or “1”)
6. If the annotator finds the tweet to be inconclusive, they should select the code that they feel is most likely correct and make notes in the Notes columns about what was problematic in classifying the tweet.

The section “[Positive Cases](#_Positive_Cases)” will define and provide examples of tweets that indicate that the user has been diagnosed with COVID-19 and should be coded as “1”. The “[Negative Cases](#_Negative_Cases)” will define and provide examples of tweets that are not indicative of the user having been diagnosed with COVID-19 and therefore should be coded as “0”. The “[Special Cases](#_Special_Cases)” section will discuss examples of tweets that may be challenging to annotate and their resolution.

## Positive Cases

In order for a tweet to be classified as a positive case, the following two criteria must be met:

1. The user is discussing their own diagnosis of COVID-19, AND
2. The user has received a clinical diagnosis of COVID-19

Each criterion is defined as follows:

### User Self-Report

The following are situations and examples of a user self-reporting their own COVID-19 diagnoses:

1. The user is stating that they are the one who has been diagnosed with COVID-19 which may be indicated through the use of first-person pronouns.

Examples:

- 1. *On my isolation day (because* ***my*** *Covid 19 test is positive) I decided wildly and bought a new guitar. Last year I bought new a ukulele again in April. I'm looking forward to what will happen next year.🤷🏻‍♀️*
  2. *@<user> Congrats! I’m so upset because they called me to schedule my vaccine 2 days after* ***I*** *was diagnosed with COVID, and now I have to wait another 2 months. But I’ll be in the front of the line then (right after I donate convalescent plasma).*

In both these examples, the use of first person pronouns indicate that the user is self-reporting a COVID diagnosis or positive test.

1. It can be inferred that the user is discussing their own COVID-19 diagnosis even though they may not have used a first person pronoun
   1. *Heya! Was supposed to start streaming this week, but got diagnosed with COVID Pneumonia today. Took all precautions all this time, but somehow still caught it. Shall be back to streaming as soon as I am feeling better. You all take care of yourselves and get vaccinated!*

While the user does not explicitly state “I got diagnosed with COVID Pneumonia…” there are contextual clues in the tweet that they are discussing their own diagnosis.

1. Self-reports of past diagnosis of COVID-19 should be coded as positive

Examples:

- 1. *I am B+(positive) blood group from Kolkata. Please connect with me for plasma donation.* ***I*** *was tested covid positive in July 2020... #PlasmaDonation #KolkataCovid*
  2. *When I woke up, the first thing that I saw was the news about Tawan. 🥺 We had the same symptoms when* ***I*** *tested positive for COVID last November 2020. It’s kinda hard to breathe if you have cough though. I hope his X-Ray results would turn normal. Stay strong. #GetWellSoonTaytawan*

1. Any reports of other’s positive COVID-19 diagnosis would only be classified as positive if the user has mentioned that they too have been diagnosed.
   1. *@<User> I have seen your discussion with DR Venkat. My wife and* ***I*** *just tested positive for covid. Can you ask if we now have immunity and whether we should get or skip vaccine? And how long should we wait if we need to get it?*

### Clinical Diagnosis

For the purpose of this study, we will consider the following status mentions as confirmatory evidence that the user has, or has in the past, been clinically diagnosed with COVID-19:

- **Diagnosed**: the user states they have been diagnosed with COVID-19
  - *@<user> @<user> I was literally diagnosed with covid the day before launch... it was like the Playstation gods were looking upon me.*
  - *@<user Great news Maribella, I am due for my FIRST Moderna on this coming Thursday. I was diagnosed with Covid in January, received the antibody treatment the next day, and now my 3 month wait is almost over.*
- **Hospitalized**: the user states they were hospitalized due to COVID-19
  - *Caught Covid, spent two months in the hospital, got trached and now spending my days looking for affordable home care so my kids can go to school and work. It’s an oxymoron - Affordable Healthcare.*
  - *@<user> Hey Briggs! I can do ya one better! Was in hospital with covid phenimina! Have to have heart surgery soon! Bad shape! Good luck with a new job pal! Thinking of you! hugs Cyndra*
  - *Brilliant news👏 I would have my like yesterday - having been in hospital for 3 weeks and beat COVID 19 - I would encourage every to attend and have the jab - any please don’t waste the vaccine and NOT attend. 💙*

All these examples also show the use of inference to determine the proper classification. It is not always the case that the user will explicitly state that they are referring to themselves by the use of “I” or “my”, however, we can infer self-reporting from other clues in the tweet.

- **Tested Positive**: the user states that they have tested positive either for COVID-19 (viral, or antigen, test) or COVID antibodies (antibody or serology test) indicating that they have had a past infection
  - *I've tested positive for corona two times in the last 6 months god really hates me*
  - *Me with 101 fever and positive covid test: what if my kids don’t do the work with their sub? 🥲*
- *@<User> @<User> I had Covid-19 in Feb. I tested positive for antibodies in May. If I was reexposed I would speculate I would be asymptomatic since my only symptoms were my immune reacting to the virus then beating it on its own.No vaccine thanks don't need it. Too many need it before me anyway*
- *@<user> This is my case - I had covid last March, had a positive antibody test in October to confirm I had it, received 1st vax in Jan and about to receive 2nd dose next week. People working in healthcare/social care settings received the vaccine as a priority group.*
  - *@<user> A point I’ve pondered a lot lately, I had Covid19 at the beginning of the lockdown in late March, I recently had bloods done &amp; requested an antibody test which proved positive 7 months after infection, but my GP insists I have not got immunity, this is why I’ll decline a vaccine!*

It is not necessary that the user explicitly states that they “tested positive for COVID-19”, a mention that the user “tested positive” can be inferred that they are referring to COVID-19

- *i was shit talking corona and got a sms of the healthcare center not even second later telling me my test was positive..* [*https://t.co/v4eTCVdu0h*](https://t.co/v4eTCVdu0h)
- *I just checked my covid test results and Im positive 🙃*

In these examples, it can be inferred from the beginning of the tweet that user is referencing COVID in relation to the positive test they mention.

## Negative Cases

In general, any tweet that does not meet the criteria to be classified as a [Positive Case](#_Positive_Cases) will be classified as a Negative Case (code = “0”). In this section, we will describe some explicit exclusion criteria that would qualify a tweet as a Negative Case.

### Negative or Unreported Testing Results

The user states that their test results came back negative:

- *on a positive note im making penne alla vodka today and my covid test was negative*
- *@<user> @<user> @<user> Okay I was just curious cause I had the COVID test around December and tested negative and both my roommates tested positive. Prob just a weird thing cause I’m guessing I had antibodies*

**OR**, the user states they have been tested but either have not yet received the results or do not report the results:

- Waiting for COVID test results. Need to know if I can go into the office Monday. Waiting. Of coursemy employer didn’t recommend me get tested. I didn’t really matter. I’m a worker ant. Actually my employer knows someone I. The office got a COVID positive. My said nothing
- Just got my Covid test because my boss let a girl come into work who was exposed without having her test results back and now exposed everybody because she came back positive… *TONGUE POP* Anyways just got my test and the pharmacist was DADDY as F***.. I might go back..🥵
- 10 mins before I know my covid test results......positive or negative? Cuz I been SICK !
- *if i test positive for covid (which is likely) in ordering myself a feast no lie. i deserve it*

### Report **Not** of User’s Diagnosis

The tweet is reporting a diagnosis but it is not a self-report. These include reports of family members, co-workers, general mentions, etc:

- My stepson Daren has Multiple Sclerosis (MS). He was just admitted to the hospital with COVID, it has gone to his lungs. If you pray, please pray for Daren. We’re scared for him. He cannot receive certain COVID treatments due to MS. #CoronavirusPandemic
- Boyfriends covid test came back positive and I was in close contact with him yesterday 😭
- My office is the worst I guess, one tests positive and they hide it, leaving other employees to ponder who it was, where and when that person tested positive. You have to overthink it everyday if you will get covid, it's not healthy anymore.

### Clinical Diagnosis not Reported

In instances where the user states that they have or had COVID-19, or think they have COVID-19, but provide no statements that they have been diagnosed with COVID-19, the tweet should be coded as a negative case:

- - *Got the first dose of the vaccine yesterday and I feel worse now than when I actually had covid. I was so sick last night I nearly passed out and went to the hospital. Hate this 😩*
  - *@<user> It's true. My family and I battled COVID without going to the hospital because we continued to move around, get fresh air, and prop ourselves up at night instead of lying down.It's the most natural way to help your body power through!*
- *@<user> @<user> Trust me I’m a woman. If I was as sick as she is stating NO WAY would I put on makeup and do my hair. Had Covid, looked like hell for a week. Was never hospitalized.*
- *i don't know i think i have covid but my country is in the worst situation ever so i can't go to the hospital until i get worse, i'll just have to survive*

In these examples, the users state that the “had COVID” but do not provide within the tweet any evidence of diagnosis, because of this the tweet would be considered a negative case.

Similarly, if the user states that they had symptoms of COVID but do not provide any explicit or implicit evidence that they were clinically diagnosed, the tweet should be coded as a negative case:

- - *@<user> Guys I think we are not doing enough because last month I had Covid related symptoms and never went to hospital but people have been sick without knowing*
- *DO NOT go to TMC ER. These mfers had me in the ER for 4 hours, treating me as a kidney stone patient when i went in with Covid/Flu symptoms. Thanks for the stupid bill &amp; another visit to an urgent care🙄*
- *You guys, #COVID19 is NO JOKE! Ive been sick for a week and I have: - difficulty breathing - inability to concentrate - intense fatigue - no taste or smell - waves of fever - nausea If I had any pre-existing conditions, I would have been hospitalized long ago*!

## Special Cases

In this section, we will review some instances where the correct annotation may be unclear. The section is divided into two, the first part will provide explanations and examples where the tweets would be considered a Positive Case and the second part will provide explanations and examples of Negative Case.

This section will be updated during the annotation project, as needed, as new examples are found.

### Special Cases – Positive

#### Conflicting Test Results Reported

Tweets in which the user reports both positive and negative test results should be classified as positive (code= “1”):

- *Covid testing nothing but a giant Snotfest. Watch this video. I tested 4 times both rapid and PCR 4 times rapid was positive 3 times PCR negative then within 5 days tested again PCR and came back negative. Im happy to forego any public domain requiring vacc. Those ppl are sick.*
- *@<user> @<user> They really aren't! I had a rapid one test negative, two days later test positive with a 48hr test then hospitalised with covid phenomena!*
- *@<user> @<user>* I was tested covid positive 20 before. Now I am negative and I am planning to travel. Will I be exempted from 3 days govt hotel

### Special Cases – Negative

#### Quoted or Reported Speech

Quoted or reported speech are instances where the person is repeating what someone else has said. This may be stated exactly as the other person said it (quoted speech) or may be as a summary or paraphrase (reported speech). In Twitter, these are sometimes identified as retweets (RT), which are easily identified and have been removed from our collection. However, some users repost information with using the retweet feature in Twitter.

Tweets which meet the criteria for inclusion as a Positive case but are not reports of the users own experience should be coded as a negative case.

Sometimes it will be easy to identify these as the user posts the quoted or reported speech with quotations marks:

- *“After experiencing mild symptoms, I’ve just tested positive for COVID.," @RahulGandhi tweeted.#COVIDSecondWaveInIndia#Covid19#RahulGandhi*
- *27 March 2020 - "I‘ve tested positive. Thankfully my symptoms are mild and I’m working from home &amp; self-isolating"29 April 2021 - Takes experimental covid vaccine completely unnecessary (for him)*

In these examples, the statements ***I’ve just tested positive for COVID*** and ***I‘ve tested positive*** would normally be indicative of the Positive class, however, since they are quoted speech and not a self-report of COVID-19 by the person who posted the tweet, they would be classified as Negative.

Other examples of this may not be as clear, however, there may be other clues in the tweet. For example:

*I Was The First Patient Hospitalized Due To COVID-19 https://t.co/kAwkcQRTIN* ***via*** *@Inside My Mind**

(*tweet modified to show example)

While there are no quotation marks used, there are some contextual clues that this tweet is reported speech and not a self-report by the person who posted the tweet. First is the use of the term “via” followed by an account name. This indicated that the user has gotten the information from another source. Second, the capitalization use is indicative of a title and the following of the text with a link may further indicate that this is a title to another written piece and not the users self-report.

There may be other instances where it just “seems” like the user is not stating a self-report. In these cases, if in the judgement of the annotator, the tweet represents quoted or reported speech, it should be classified as a Negative case. The annotator should make a notation of why they decided this in the Notes column.

#### Positive Antibody Test Due to Vaccine

There is some evidence that a person vaccinated with the COVID-19 vaccine will have a positive antibody test. If the user is attributing their positive antibody results to having been vaccinated rather than a past COVID-19 infection, the tweet should be classified as a Negative case:

*But.. here we are!My test results after 14 and a half days from my 2nd Pfizer #CovidVaccine dose: Negative for Coronavirus, Positive for Antibodies!Never got Covid. Never gave it to anyone. And I'm alive!*
